# A Modified Enhanced Recovery After Surgery Protocol Versus Standard Care in Pediatric Major Gastrointestinal Surgery: A Randomized Controlled Trial

**DOI:** 10.7759/cureus.110322

**Published:** 2026-06-05

**Authors:** Andi Ade Wijaya Ramlan, Kshetra Rinaldhy, Rahendra Rahendra, Raihanita Zahra, Komang Ayu Ferdiana, Rizky Amaliah, Hardian Gunardi, Wulan Ayudyasari, Niken Wahyu Puspaningtyas, Christopher Kapuangan, Arif Hari Martono Marsaban

**Affiliations:** 1 Department of Anesthesiology and Intensive Care, Faculty of Medicine, Universitas Indonesia, Dr. Cipto Mangunkusumo Hospital, Jakarta, IDN; 2 Department of Surgery, Faculty of Medicine, Universitas Indonesia, Dr. Cipto Mangunkusumo Hospital, Jakarta, IDN; 3 Department of Child Health, Faculty of Medicine, Universitas Indonesia, Dr. Cipto Mangunkusumo Hospital, Jakarta, IDN

**Keywords:** carbohydrate loading, early mobilization, enhanced recovery after surgery, intraoperative glucose, length of hospital stay, pediatric anesthesia, pediatric eras protocol, pediatric gastrointestinal surgery, perioperative care pathway, randomized controlled trial

## Abstract

Background

Perioperative management in pediatric major gastrointestinal surgery often involves prolonged fasting, delayed mobilization, and invasive monitoring, which may increase stress responses and slow recovery. Enhanced recovery after surgery (ERAS) pathways are well established in adults but require pediatric adaptation. This study evaluated a modified ERAS protocol versus standard care in pediatric patients undergoing major gastrointestinal surgery.

Methodology

This single-blind randomized controlled trial enrolled pediatric patients aged two months to five years undergoing major gastrointestinal surgery. Participants were randomized to a modified ERAS group (n = 40) or standard care group (n = 42). Outcomes included time to first oral nutrition, time to first mobilization, time to urinary catheter removal, length of hospital stay, and intraoperative glucose and electrolyte levels.

Results

Early mobilization was significantly faster in the modified ERAS group (p = 0.014), and length of hospital stay was shorter (p = 0.018). The modified ERAS group demonstrated lower intraoperative glucose levels at later intraoperative timepoints (timepoint 2, p = 0.047; timepoint 3, p = 0.008). There were no significant differences in time to first oral nutrition, urinary catheter removal, or intraoperative electrolyte levels between groups.

Conclusions

A modified ERAS protocol in pediatric major gastrointestinal surgery was associated with earlier mobilization, shorter hospitalization, and improved intraoperative glucose stability compared with standard care. Adoption of pediatric-adapted ERAS pathways may improve perioperative outcomes in this population.

## Introduction

Children undergoing major gastrointestinal surgery are particularly vulnerable to perioperative stress and delayed recovery due to traditional practices such as prolonged fasting and delayed mobilization. Enhanced recovery after surgery (ERAS) offers a multidisciplinary, evidence-based approach to improve perioperative outcomes. Initially developed for adults, ERAS protocols have demonstrated benefits in adult surgical recovery but remain underutilized in pediatric surgery due to physiological differences and limited pediatric-specific standardization [1]. Observational studies in pediatric colorectal surgery using ERAS protocols have shown benefits similar to those observed in adults, including reduced postoperative complications, shorter hospital stays, earlier enteral nutrition, and lower healthcare costs [2,3].

Adapting ERAS protocols for pediatric patients remains challenging due to developmental, physiological, and clinical differences. Evidence regarding the value of ERAS in children undergoing major abdominal surgery remains limited, and not all adult ERAS elements can be directly applied to pediatric patients [1,4]. Therefore, modifying ERAS protocols to suit pediatric characteristics has become increasingly essential.

Key components of optimized perioperative pathways include preoperative counseling, limited preoperative fasting, optimal anesthesia, minimally invasive techniques, early postoperative oral nutrition and mobilization, and nonroutine use of surgical drains and tubes. ERAS challenges conventional perioperative protocols to optimize inpatient care and minimize patient discomfort [5,6].

Dr. Cipto Mangunkusumo Hospital is a leading pediatric surgery center in Indonesia, performing more than 3,000 surgeries annually with high patient and procedural complexity. While ERAS protocols in children have been implemented for less complex procedures, evidence for major abdominal surgery remains limited. Differences in perspectives among pediatric surgeons, anesthesiologists, and pediatricians have contributed to the lack of an ERAS pathway for major abdominal surgery at the institution. Despite supportive evidence, resistance persists due to entrenched conventional perioperative practices, limited dissemination of ERAS principles, and the need for strong multidisciplinary coordination and administrative support for successful implementation [7,8].

This study aimed to develop a multidisciplinary ERAS protocol tailored for pediatric patients undergoing major gastrointestinal surgery and to evaluate its effectiveness compared with standard care at the institution.

## Materials and methods

Study design and participants

The study is a single-blind randomized clinical trial conducted in the elective operating room at Dr. Cipto Mangunkusumo Hospital, Jakarta, Indonesia. This study aimed to compare the postoperative outcomes of pediatric patients who received the ERAS protocol before elective surgery versus those who received the standard protocol. This study obtained ethical approval from the Research and Ethics Committee, Faculty of Medicine, Universitas Indonesia, and was registered at ClinicalTrials.gov (NCT06981572). The estimated sample size required for each group in this study was calculated based on the sample size formula for unpaired categorical/numerical analytical research. It was determined that a minimum of 90 pediatric patients aged two months to five years with American Society of Anesthesiologists (ASA) physical status 1, 2, and 3, scheduled for elective major abdominal surgery under general anesthesia, were included as study participants. Patients with cyanotic heart disease, metabolic or endocrine abnormality, decreased mental status, or severe cognitive dysfunction were excluded. Patients undergoing re-laparotomy due to previous surgical complications were also excluded. Informed consent to participate in the study was obtained from the parents or caregivers in accordance with hospital policy. This randomized controlled trial was designed and reported in accordance with the CONSORT 2025 guidelines [9]. Surgical approach was categorized as open (standard) versus minimally invasive surgery. Surgical approach was included among intraoperative characteristics compared between groups.

Preparations

A focus group discussion was conducted before study initiation to develop a modified ERAS protocol suitable for pediatric patients. The multidisciplinary team consisted of anesthesiologists, pediatric surgeons, pediatricians, and perioperative nurses, who reviewed current literature and adapted established adult ERAS protocols to ensure feasibility and applicability in routine pediatric perioperative care. The discussion resulted in the modified ERAS protocol for preoperative, intraoperative, and postoperative care (Table 1).

**Table 1:** Core elements of the modified enhanced recovery after surgery protocol. ASA: American Society of Anesthesiologists; myPAS-SF: modified Yale Preoperative Anxiety Scale–Short Form; PONV: postoperative nausea and vomiting; ETCO₂: end-tidal carbon dioxide; ECG: electrocardiogram; NIBP: noninvasive blood pressure; kgBW: kilogram body weight

Core elements	Modified ERAS
Preoperative	
Counseling	Using a pocket book. Provided education regarding procedures to be performed, planned anesthesia and medications (including side effects), fasting preparation, nutritional preparation, physical activity, and postoperative goals
Optimization of medical condition	Preoperative assessments include ASA physical status, nutritional status, comorbidities, anxiety and PONV risk assessment using myPAS-SF and Apfel scoring, respectively, and appropriate treatment based on the underlying condition
Bowel preparation	No
Fasting guidelines	Modified preoperative fasting: Start Carbolyte 6 hours before surgery (or 12 hours for the first schedule; 24 mL/kgBW for 6 hours), ending 1 hour before surgery (3 mL/kgBW). Fasting solid food or formula milk for 6 hours. Fasting breast milk for 4 hours. Whenever possible, all diets should be taken orally rather than via an NGT
Preemptive non-opioid analgesics	Oral ibuprofen 15 mg/kgBW, 12 hours before surgery and 1 hour before surgery
Prophylactic antibiotics	Administer empiric antibiotics for infection prophylaxis within 60 minutes before surgical incision
Intraoperative	
Anesthesia protocols	Standardized anesthesia technique with short- to moderate-acting opioids during induction. Regional anesthesia: caudal, epidural, or peripheral block (unless contraindicated). Standard monitoring: NIBP, pulse oximeter, ETCO₂, oral or nasopharyngeal temperature, and ECG
Fluid management	Goal-directed balanced crystalloid solution with 1% dextrose for maintenance, and balanced crystalloid solution for replacement as needed
Minimally invasive surgery	As needed and available, following surgical decision
Postoperative	
Avoiding an intraperitoneal drain	Following surgeon’s decision, remove as soon as patient condition is ready
Avoiding routine nasogastric tube	Following surgeon’s decision, remove as soon as patient condition is ready
Hypothermia prevention	Yes, standardized protocol
Multimodal protocols for PONV	Yes, standardized protocol
Pain management	Following anesthesia and surgical team guidance using hospital acute pain protocol (paracetamol 15 mg/kg every 4 hours)
Early mobilization	Yes
Early oral nutrition	Yes

Educational materials were developed for children and their parents or caregivers to support preoperative counseling. A carbohydrate- and electrolyte-containing solution was prepared for preoperative carbohydrate loading, which is widely accepted by pediatric patients in our hospital [10].

Interventions

Once the patient was deemed ready for surgery, the pediatric surgeon assessed eligibility based on the inclusion criteria. If eligible, the patient was flagged as a potential study subject in the electronic medical record, and the research assistant was notified for follow-up. The study protocol commenced upon the patient’s hospital admission. Participants were randomized 1:1 into the ERAS (n = 45) or standard care group (n = 45) using a simple randomization method with a computer-generated sequence (randomizer.org), with allocation concealed in sealed opaque envelopes. Patients assigned to the ERAS group received the modified ERAS protocol in accordance with Table 1. Meanwhile, standard care consisted of preoperative six hours of solid food fasting, two hours of no oral liquid intake, ibuprofen one hour before surgery, propofol and inhalation maintenance (sevoflurane) during surgery, and nonsteroidal anti-inflammatory drugs adjusted to the patients’ needs. No specific guideline was followed in the standard care group, which was tailored by the clinicians’ (anesthesiologists and pediatric surgeons’) judgement. Outcome assessors and data analysts were blinded to group assignments throughout the trial and had no access to the allocation sequence or intervention details. Due to the nature of the perioperative pathway intervention, treating clinicians and bedside care teams could not be blinded to group allocation. The ERAS group received perioperative management based on a tailored ERAS protocol, while the standard care group received conventional perioperative care. The modified ERAS pathway was implemented as part of perioperative care with coordination by the study team to support protocol delivery and documentation; clinical decisions remained under the responsibility of the treating surgical and anesthesia teams, guided by institutional standards and each patient’s clinical condition.

Outcomes

Primary outcomes included length of hospital stay and time to first mobilization. Secondary outcomes included time to first oral nutrition, urinary catheter removal, intraoperative glucose and electrolyte levels, and perioperative anxiety. The intraoperative parameter monitoring was measured five times, starting from the induction time (timeframe 1), over the interval of two hours for each measurement (300 minutes after induction at timeframe 5).

Statistical analysis

Data were analyzed using SPSS version 20 (IBM Corp., Armonk, NY, USA). The normality of continuous variables was assessed using the Shapiro-Wilk test. For group comparisons, independent t-tests were used for normally distributed continuous variables, while the Mann-Whitney U test was applied for non-normally distributed data. Categorical variables were analyzed using the chi-square test. A p-value <0.05 was considered statistically significant. Multivariable analysis using analysis of covariance (ANCOVA) was conducted to further control for potential confounding variables. ANCOVA was selected because the primary objective was to evaluate the association between ERAS implementation and postoperative outcomes while adjusting for important baseline and perioperative covariates that could independently influence recovery. Variables included in the model were selected based on their clinical relevance and potential impact on postoperative recovery, including surgical invasiveness and preoperative laboratory parameters.

## Results

**Figure 1:**
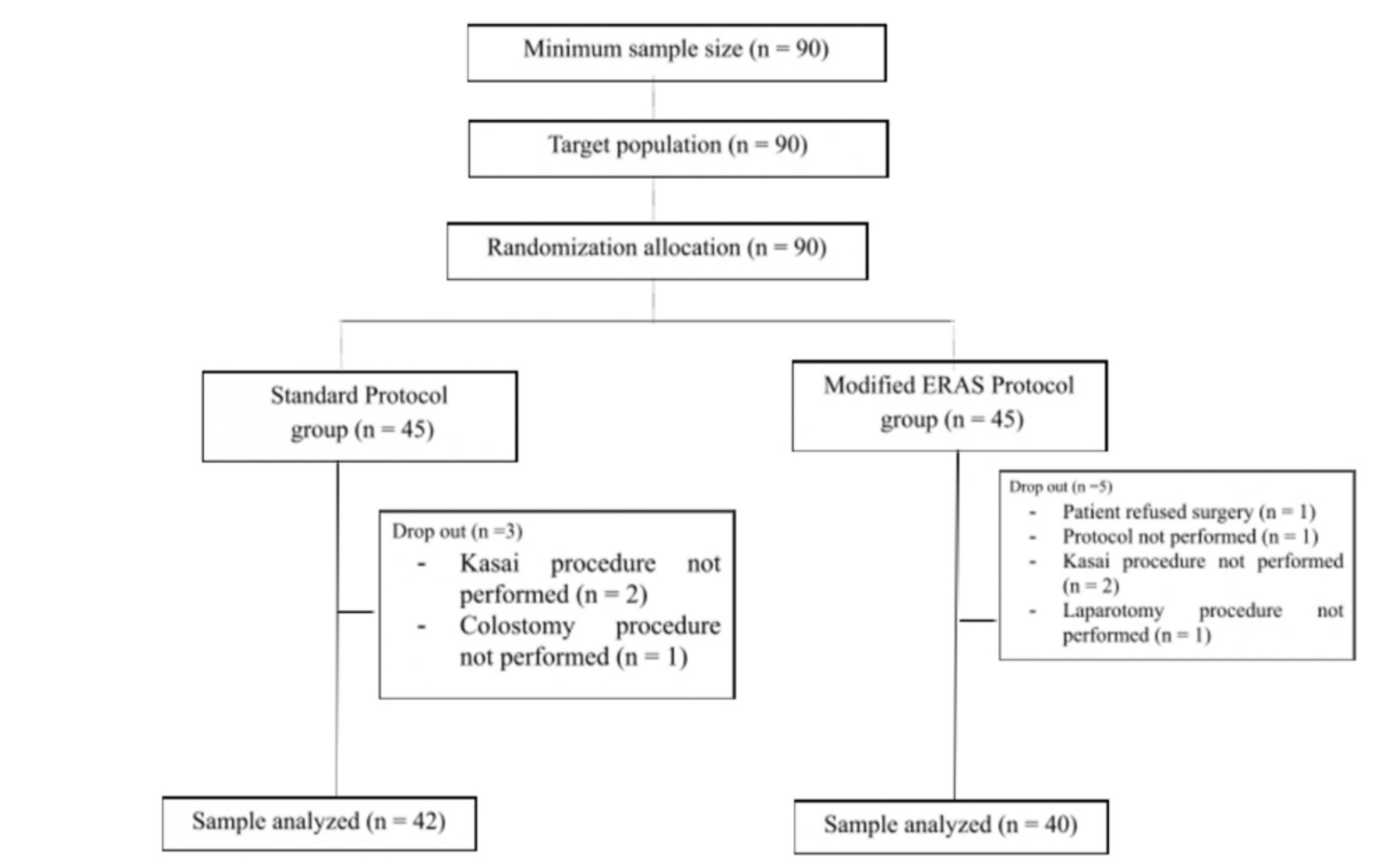
CONSORT flow diagram. CONSORT flow diagram of participant enrollment, allocation, follow-up, and analysis. ERAS: enhanced recovery after surgery

A total of 90 patients were randomized into two groups: 45 allocated to the modified ERAS group and 45 to the standard protocol group. Following the exclusion of eight dropouts, 82 patients were included in the final analysis (Figure 1).

Baseline characteristics were generally comparable between groups, except for a significantly higher prevalence of prior surgery in the ERAS group compared to the control group (75% vs. 47.6%, p = 0.021), as detailed in Table 2.

**Table 2:** Baseline characteristics of patients included in the study. Anxiety was defined as mYPAS-SF ≥30. ERAS: enhanced recovery after surgery; ASA: American Society of Anesthesiologists; PONV: postoperative nausea and vomiting; mYPAS-SF: modified Yale Preoperative Anxiety Scale–Short Form

Variables	Total	Intervention group		P-value
		Modified ERAS protocol group (n = 40)	Standard protocol group (n = 42)	
Sex				0.682
Male	48 (58.5)	22 (55)	26 (61.9)	
Female	34 (41.5)	18 (45)	16 (38.1)	
Age (months)	14 (5–27)	13 (7–27)	14 (3–38)	0.799
Body weight	8.42 ± 3.18	8.36 ± 2.81	8.47 ± 3.53	0.882
Body height	71 ± 17.13	71.06 ± 15.99	70.94 ± 18.34	0.397
ASA status				1.000
ASA 2	81 (98.8)	40 (100)	41 (97.6)	
ASA 3	1 (1.2)	0 (0)	1 (2.4)	
Nutritional status				0.366
Severe malnutrition	2 (2.4)	1 (2.5)	1 (2.4)	
Moderate malnutrition	8 (9.8)	2 (5)	6 (14.3)	
Normal	72 (87.8)	37 (92.5)	35 (83.3)	
Comorbidities				0.255
Yes	15 (18.3)	5 (12.5)	10 (23.8)	
No	67 (81.7)	35 (87.5)	32 (76.2)	
Risk of PONV				1.000
Low risk	75 (91.5)	37 (92.5)	38 (90.5)	
Moderate risk	7 (8.5)	3 (7.5)	4 (9.5)	
Prior surgery				0.021
Yes	50 (61)	30 (75)	20 (47.6)	
No	32 (39)	10 (25)	22 (52.4)	
mYPAS-SF score	22.92 (22.92–33.33)	22.92 (22.92–29.17)	22.92 (22.92–33.33)	0.627
Incidence of anxiety				0.266
Yes	22 (26.8)	8 (20)	14 (32.3)	
No	60 (73.2)	32 (80)	28 (66.7)	
Invasiveness				0.082
Standard	67 (81.7)	30 (36.5)	37 (45.1)	
Minimal	16 (19.5)	11 (27.5)	5 (11.9)	

Intraoperative surgical approach was recorded as open (standard) versus minimally invasive. Minimally invasive surgery was performed in 11/40 (27.5%) patients in the modified ERAS group versus 5/42 (11.9%) in the standard care group. We observed no significant difference between the ERAS and standard care group.

The ERAS group demonstrated earlier mobilization compared with the standard care group (2 ± 0.86 vs. 3 ± 0.83 days; p = 0.014), corresponding to a mean difference of −1.00 days (95% confidence interval (CI) = −1.37 to −0.63; Hedges g ≈ −1.17). Length of hospital stay was also shorter in the ERAS group (8 ± 2.67 vs. 10 ± 3.82 days; p = 0.018), with a mean difference of −2.00 days (95% CI = −3.44 to −0.56; Hedges g ≈ −0.60). No statistically significant differences were observed between groups in time to oral intake initiation, urinary catheter removal, or drain removal (Table 3).

**Table 3:** Postoperative recovery outcomes. Values are presented as mean ± standard deviation (SD). P-values were calculated using an independent samples t-test. *: Statistically significant. ERAS: enhanced recovery after surgery

Variables	Total	Intervention group		P-value
		Modified ERAS protocol group (n = 40)	Standard protocol group (n = 42)	
Time to initiation of oral intake (hours)	41 ± 27	41 ± 27.41	43 ± 26.89	0.691
Time to drain removal (days)	6 ± 2	7 ± 2.04	6 ± 2	0.738
Time to first mobilization (days)	2 ± 1	2 ± 0.86	3 ± 0.83	0.014*
Time to urinary catheter removal (days)	3 ± 2	3 ± 2.03	4 ± 3.86	0.484
Length of hospital stay (days)	9 ± 3	8 ± 2.67	10 ± 3.82	0.018*

**Figure 2:**
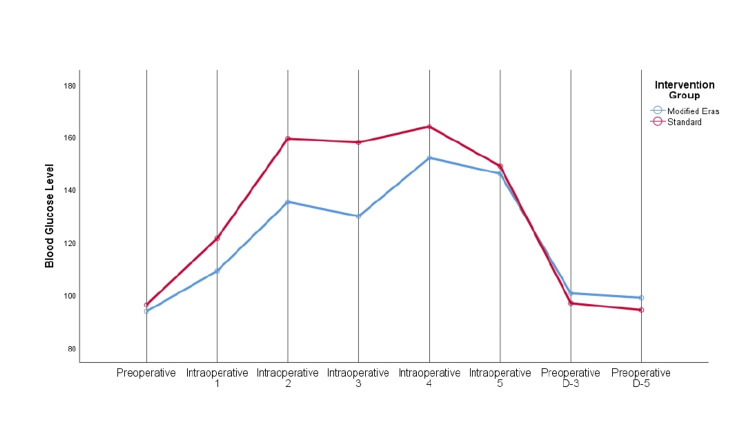
Intraoperative glucose levels by group. Line graph of intraoperative glucose measurements at predefined timepoints in the enhanced recovery after surgery and standard care groups.

No significant differences were observed in pre- or postoperative electrolyte levels. However, intraoperative glucose levels were significantly lower in the ERAS group at timepoints 2 and 3 (p = 0.047 and p = 0.008, respectively), indicating improved glycemic control (Table 4, Figure 2). At intraoperative timepoint 2, glucose was lower in the ERAS group by −24.0 mg/dL (95% CI = −46.2 to −1.9; Hedges g ≈ −0.47), and at timepoint 3 by −28.0 mg/dL (95% CI = −44.5 to −11.5; Hedges g ≈ −0.74).

**Table 4:** Perioperative glucose and electrolyte profiles. Values are presented as mean ± standard deviation (SD). P-values were calculated using an independent samples test; *: statistically significant. Timepoints refer to predefined intraoperative measurement timepoints. ERAS: enhanced recovery after surgery

Variables	Total	Modified ERAS protocol group (n = 40)	Standard protocol group (n = 42)	P-value
Serum sodium level (mmol/dL)				
Preoperative	135.74 ± 2.54	136.05 ± 2.04	135.45 ± 2.94	0.286
Postoperative	135.72 ± 2.90	135.76 ± 2.96	135.68 ± 2.88	0.911
Serum potassium level (mmol/dL)				
Preoperative	5.09 ± 5.49	4.45 ± 0.55	5.71 ± 7.56	0.299
Postoperative	4.23 ± 0.55	4.18 ± 0.52	4.29 ± 0.58	0.384
Serum chloride level (mmol/dL)				
Preoperative	104.26 ± 3.93	104.11 ± 3.70	104.41 ± 4.17	0.730
Postoperative	101.54 ± 4.24	101.04 ± 3.93	101.98 ± 4.45	0.336
Blood glucose level				
Preoperative	95.01 ± 20.84	93.73 ± 21.38	96.24 ± 20.50	0.588
Intraoperative				
Timepoint 1	115.45 ± 42.05	109.13 ± 36.42	121.48 ± 45.41	0.185
Timepoint 2	148.08 ± 52.28	135.26 ± 37.41	159.30 ± 60.91	0.047*
Timepoint 3	146.72 ± 40.17	129.83 ± 33.66	157.85 ± 41.15	0.008*
Timepoint 4	160 ± 55	152.00 ± 28.32	164.85 ± 65.03	0.589
Timepoint 5	147 ± 39	146.00 ± 35.75	148.89 ± 43.35	0.879
Postoperative				
Day 3	98.67 ± 18.55	100.60 ± 18.48	97.92 ± 18.65	0.387
Day 5	96.30 ± 17.93	99.93 ± 15.53	94.24 ± 19.56	0.282

Only 72 of the 82 samples could be assessed for Pediatric Anesthesia Emergence Delirium (PAED) scores because 10 patients remained intubated post-surgery. There was no difference in PAED scores (median = 5 in both groups, p = 0.770) or incidence of emergence delirium between the two treatment groups (Table 5).

**Table 5:** Incidence of emergence delirium, PONV, and postoperative pain in the intervention groups. Values are presented as median (IQR) for continuous variables (Mann–Whitney U test) and n (%) for categorical variables (chi-square test). ERAS: enhanced recovery after surgery; PAED: Pediatric Anesthesia Emergence Delirium; FLACC: Face, Legs, Activity, Cry, Consolability; PONV: postoperative nausea and vomiting; IQR: interquartile range

Variables	Total	Intervention group		P-value
		Modified ERAS protocol group (n = 40)	Standard protocol group (n = 42)	
PAED score	5 (4–6)	5 (4–6)	5 (4–6.5)	0.770
Emergence delirium				1.000
Yes	9 (12.5)	5 (13.9)	4 (11)	
No	63 (87.5)	31 (86.1)	32 (88.9)	
FLACC score (recovery room)	1 (0–2)	1 (0–3)	1 (0–2)	0.819
Pain 24 hours postoperatively				0.370
Yes	20 (24.2)	12 (30)	8 (19)	
No	62 (75.6)	28 (70)	34 (81)	
Incidence of PONV				0.477
Yes	8 (9.8)	5 (12.5)	3 (7.1)	
No	74 (90.2)	34 (87.5)	39 (92.9)	

There was no difference in immediate postoperative pain incidence between groups (Face, Legs, Activity, Cry, and Consolability (FLACC) >3: 30% vs. 19%, p = 0.370). No opioid was administered postoperatively in either group, and pain management followed hospital guidelines for acute pain management.

Further multivariate analysis was conducted to account for important covariates in this study (surgical invasiveness, surgical length, intubation, etc). Using ANCOVA, ERAS was found to have an important interaction with invasiveness (p = 0.037). In minimally invasive patients, ERAS appeared to have higher efficacy in reducing length of stay (9.66 ± 4.3 days in the standard protocol group vs. 5.44 ± 1.59 days in the ERAS group) compared to a more invasive group (9.37 ± 3.8 days in the standard protocol group vs. 8.56 ± 2.5 days in the ERAS group). Several covariates affected this result, including operation time and preoperative potassium and blood sugar level (p < 0.05). We found no similar difference in mobilization time, urinary catheter removal, oral intake initiation, and in-operation serum biomarkers (p > 0.05), nor influence on this effect by important covariates (first operation, nutritional status, use of medical devices (catheter/nasogastric tube/intubation), postoperative laboratory measurements, and postoperative ibuprofen).

## Discussion

This randomized clinical trial evaluated the implementation of a modified ERAS protocol in pediatric patients undergoing major abdominal gastrointestinal surgery at the institution. Compared with the ERAS standard in the consensus guidelines from the ERAS Society Recommendation [11], our ERAS protocols involved standardized preoperative counseling using pocketbooks for both the children and parents, regardless of the physician performing the counseling. The anesthesia technique and intraoperative pain management standardization was only for monitoring and fluid maintenance. Narcotics administration was not standardized and performed at the direction of the anesthesia team. Postoperative pain management was standardized in accordance with hospital guidelines for acute pain management.

This study showed that the modification of the ERAS protocol is safe and effective in promoting early postoperative recovery with significant improvements in mobilization time and hospital length of stay while maintaining normal perioperative physiological parameters. One of the most important outcomes was the significantly earlier mobilization observed in the ERAS group compared to the standard care group (mean = 2 vs. 3 days, p = 0.014). Early mobilization is a cornerstone of ERAS principles and is associated with improved pulmonary function, decreased risk of thromboembolic events, and overall shorter recovery time in pediatric patients [12-14]. In addition, the patients in the ERAS group experienced shorter hospital lengths of stay (mean = 8 vs. 10 days, p = 0.018), demonstrating that the protocol may facilitate more efficient postoperative care and potentially reduce healthcare costs [1]. The ERAS group experienced a shorter preoperative fasting time, which improved patient comfort and maintained hemodynamic stability without any relevant complications, such as intraoperative or postoperative regurgitation, aspiration pneumonia, or postoperative gastric retention [15]. Furthermore, we found that the efficacy of ERAS was seen more pronounced in minimally invasive procedures as opposed to more invasive ones.

Intraoperative glycemic control was better in the modified ERAS group, particularly at intraoperative timepoints 2 and 3, where glucose levels were significantly lower than those in the standard protocol group (p = 0.047 and p = 0.008, respectively). This result may reflect the benefit of preoperative carbohydrate loading and minimized fasting, which is known to reduce perioperative insulin resistance and attenuate the surgical stress, as evidenced by lower post-inflammatory response [9,16-19]. These findings are consistent with previous studies indicating the metabolic advantages of ERAS protocols in pediatric populations [17,20,21]. The standard group also showed a more fluctuating glucose trend on the line graph compared to the modified ERAS group (Figure 2), which may indicate that preoperative administration of oral carbohydrates and electrolyte solutions can effectively prevent a sharp increase in blood glucose levels, which may cause complications during the perioperative period [22]. The carbohydrate and electrolyte solution contains 10% glucose with additional electrolytes formulated according to the child’s daily needs [10].

No adverse outcomes were found in either group. Perioperative electrolyte levels, pain scores (assessed using FLACC), incidence of emergent delirium, and postoperative nausea and vomiting were similar in both groups, supporting the comparable safety of the modified ERAS approach. Additionally, preoperative anxiety scores (modified Yale Preoperative Anxiety Scale) were comparable (Table 2), indicating that the ERAS protocol, including shorter fasting and preoperative counselling, did not negatively impact emotional stress in children [22-24].

The study has several limitations that should be addressed. Although we recorded surgical approach (open versus minimally invasive), outcomes were not stratified by individual procedure type or complexity; future studies should consider procedure-specific stratification. In addition, intraoperative changes from the planned procedure led some patients to no longer meet the criteria for major gastrointestinal surgery, contributing to an overall dropout rate of 8.9%, which was slightly higher in the ERAS group. Baseline imbalance in prior surgical history was also observed in the ERAS group, which could increase operative difficulty (e.g., adhesiolysis) and prolong recovery; therefore, this imbalance might be expected to bias mobilization and length of stay toward worse outcomes in that arm. Despite this, the ERAS group demonstrated earlier mobilization and shorter length of stay. Nonetheless, although additional adjustment for important covariates was performed using ANCOVA, residual confounding cannot be completely ruled out because of the heterogeneity of surgical procedures and the pragmatic nature of the intervention.

Furthermore, the increased use of oral ibuprofen in the ERAS group may have contributed to better comfort and faster mobilization, introducing a potential confounder in the interpretation of recovery outcomes. Nevertheless, the results of this study provide strong preliminary evidence supporting the feasibility and clinical benefits of modified ERAS protocols in pediatric gastrointestinal surgery in a tertiary care setting in Indonesia. The results have significant implications for perioperative care in low- and middle-income countries, such as Indonesia, where optimizing resource utilization and enhancing patient outcomes are crucial.

## Conclusions

In this single-blind randomized trial, a locally adapted pediatric ERAS protocol was associated with earlier mobilization and shorter hospitalization, alongside lower intraoperative glucose levels at selected timepoints, without apparent safety concerns in measured short-term outcomes. Given procedure heterogeneity, limited clinician blinding, and the absence of adherence quantification despite additional adjustment for important covariates, these findings should be interpreted as preliminary evidence supporting feasibility and potential benefit. Larger, stratified trials with standardized complication reporting and longer-term endpoints are needed to confirm clinical impact and generalizability.
